# A Pilot Genome-Wide Association Study in Postmenopausal Mexican-Mestizo Women Implicates the RMND1/CCDC170 Locus Is Associated with Bone Mineral Density

**DOI:** 10.1155/2017/5831020

**Published:** 2017-08-03

**Authors:** Marisela Villalobos-Comparán, Rogelio F. Jiménez-Ortega, Karol Estrada, Alma Y. Parra-Torres, Anahí González-Mercado, Nelly Patiño, Manuel Castillejos-López, Manuel Quiterio, Juan Carlos Fernandez-López, Bertha Ibarra, Sandra Romero-Hidalgo, Jorge Salmerón, Rafael Velázquez-Cruz

**Affiliations:** ^1^Consorcio de Genómica Computacional, Instituto Nacional de Medicina Genómica, Mexico City, Mexico; ^2^Laboratorio de Genómica del Metabolismo Óseo, Instituto Nacional de Medicina Genómica, Mexico City, Mexico; ^3^Statistical Genetics, Biogen, Cambridge, MA, USA; ^4^Centro Universitario en Ciencias de la Salud, Universidad de Guadalajara, Guadalajara, JAL, Mexico; ^5^División de Genética, Centro de Investigación Biomédica de Occidente, IMSS, Guadalajara, JAL, Mexico; ^6^Instituto Nacional de Medicina Genómica, Mexico City, Mexico; ^7^Unidad de Vigilancia Epidemiológica Hospitalaria, Instituto Nacional de Enfermedades Respiratorias, Mexico City, Mexico; ^8^Centro de Investigación en Salud Poblacional, Instituto Nacional de Salud Pública, Cuernavaca, MOR, Mexico; ^9^Unidad de Investigación Epidemiológica y en Servicios de Salud, Instituto Mexicano del Seguro Social, Cuernavaca, MOR, Mexico

## Abstract

To identify genetic variants influencing bone mineral density (BMD) in the Mexican-Mestizo population, we performed a GWAS for femoral neck (FN) and lumbar spine (LS) in Mexican-Mestizo postmenopausal women. In the discovery sample, 300,000 SNPs were genotyped in a cohort of 411 postmenopausal women and seven SNPs were analyzed in the replication cohort (*n* = 420). The combined results of a meta-analysis from the discovery and replication samples identified two loci, *RMND1* (rs6904364, *P* = 2.77 × 10^−4^) and *CCDC170* (rs17081341, *P* = 1.62 × 10^−5^), associated with FN BMD. We also compared our results with those of the Genetic Factors for Osteoporosis (GEFOS) Consortium meta-analysis. The comparison revealed two loci previously reported in the GEFOS meta-analysis: *SOX6* (rs7128738) and *PKDCC* (rs11887431) associated with FN and LS BMD, respectively, in our study population. Interestingly, rs17081341 rare in Caucasians (minor allele frequency < 0.03) was found in high frequency in our population, which suggests that this association could be specific to non-Caucasian populations. In conclusion, the first pilot Mexican GWA study of BMD confirmed previously identified loci and also demonstrated the importance of studying variability in diverse populations and/or specific populations.

## 1. Introduction

Osteoporosis (OP) is a common skeletal disorder characterized by low bone mineral density (BMD) and microarchitectural deterioration of bones, which increases the risk of fractures with a consequent increase in morbidity and mortality [1]. Mexicans are an admixed population of European, Native American, and a small proportion of African (1–3%) ancestries [2, 3]. In the Mexican population aged ≥40 years, prevalence of osteopenia and osteoporosis in 2010 was reported to be 32.8 and 8%, respectively. Costs of managing osteopenia and osteoporosis account for up to 154.9 million USD, whereas costs due to fragility fractures reached 256.2 million USD in health care services [4]. BMD clinically serves as a diagnostic index in the assessment of osteoporosis and is the most widely used predictor of osteoporotic fractures (OF) [5]. Both genetic and environmental factors are known to influence BMD; however, twin and family studies have estimated the influence of heritability to be between 0.50 and 0.85 [6].

Similar to other complex diseases, candidate genes associated with BMD in individuals of European and Asian ancestries have been evaluated in the Mexican population. However, these studies have yielded inconsistent results due to small sample sizes and statistical power limitations [7–13], which suggest that this trait varies among ethnic groups and that other genetic factors have yet to be identified, in the Mexican population.

To date, there have been over 30 genome-wide association studies (GWASs) conducted for BMD leading to the identification of nearly 100 independent loci [14, 15]. The Genetic Factors for Osteoporosis (GEFOS) Consortium conducted the largest GWAS for BMD [16]. However, these studies have been performed mostly in European and Asian populations, and it remains unclear whether these same loci contribute to BMD in Amerindian-derived populations (i.e., Mexican-Mestizo). Given that Amerindian-derived populations have been underrepresented in a GWAS, it is critical to investigate these populations to determine the genetic variants and genes that are shared among diverse populations. The aim of this study was to perform a pilot genome-wide association analysis to identify the genetic loci that influence bone mineral density in the Mexican-Mestizo population.

## 2. Materials and Methods

### 2.1. Subjects

The study group included only women born in Mexico whose parents and grandparents identified themselves as Mexican-Mestizos. The discovery sample consisted of 420 unrelated postmenopausal women over 45 years of age that were recruited from “health workers cohort study (HWCS),” which is a long-term study of workers from the Instituto Mexicano del Seguro Social (IMSS) in Cuernavaca, Morelos (Mexico central zone), that focuses on lifestyle and chronic diseases [17]. The clinical procedures, data coding, entry, and participant follow-up practices have been standardized and validated [18].

The replication cohort consisted of 420 postmenopausal women from Western Mexico who were recruited from the outpatient clinic of the Regional General Hospital Number 110 of the IMSS in Guadalajara. Trained research doctors and nurses conducted the data collection for the demographic characteristics, smoking status, menopausal status, medical history, and medication usage using a structured questionnaire [8]. All women met the inclusion criteria (at least one year of amenorrhea, Mexican-Mestizo ancestry whose parents and grandparents had Mexican ancestry for at least three generations, and no history of metabolic disorders or chronic degenerative diseases that affect BMD). Participants in both cohorts were excluded from the study if they had an oophorectomy prior to 45 years of age, started/underwent menopause prior to 40 years of age, or had a history of medication that affects bone metabolism.

To explore frequencies in Amerindian populations, we evaluated an independent sample of 366 Amerindians from the Consortium for the Study of Genomic Diversity of the Indigenous Populations whose origin is mainly from three indigenous groups (Nahua, Totonac, and Zapotec). Only subjects who were born in their communities and speak their native language were included. Samples were previously genotyped with the Genome-Wide Human SNP 6.0 Microarray (Affymetrix, Santa Clara, CA). The Ethics Committees from all participating institutions approved the protocol, and written informed consent was obtained from all individual participants (discovery, replication, and consortium diversity) included in the study.

### 2.2. Measurement of Bone Mass Density

In the discovery sample, BMD was measured at the femoral neck (FN) and lumbar spine (LS, L2–L4) using a dual-energy X-ray absorptiometry (DXA) Lunar DPX NT instrument (Lunar Radiation Corp., Madison, WI). In the replication cohort, BMD was measured at the lumbar spine (L1–L4) and left and right femoral neck using DXA (Prodigy Advance, GE) by trained individuals according to the manufacturer's protocols. Both instruments use the same software; therefore, the measurements are reproducible and comparable. Standard calibration of the instruments was performed daily using a manufacturer-provided phantom for the spine and femoral neck. Technicians ensured that the daily variation coefficient (VC) was within normal operational standards and that the in vivo VC was lower than 1.5%. Measurements are presented in grams per square centimeter and were used to analyze the variations in BMD.

### 2.3. Genotyping and Quality Control

Out of the 420 postmenopausal women in the HWCS (discovery sample), DNA was available from 420, all of whom were genotyped using the Human CytoSNP-12 DNA v2.1, which included 300,000 SNPs. Standard quality control (QC) measures involved the removal of duplicates and first- or second-degree relatives based on an identity-by-descent (IBD) analysis computed by the PLINK program [19]. This analysis was conducted only in the discovery sample (Morelos cohort). We only included unrelated individuals with a 97% call rate and gender concordance. Furthermore, we excluded SNPs that had an allele frequency < 5% and call rate < 95%. After QC filters, 411 women were included in the association study, with a total of 225,635 autosomal SNPs for analysis. Across the 50 duplicated samples, the genotype concordance exceeded 99.0%. Seven SNPs were genotyped in the replication sample consisted of 420 women, using commercial predesigned TaqMan probes (Applied Biosystems, Foster City, CA, USA), according to previously described methods [11]. Information on the parental frequencies and genotype data for a panel of 96 ancestry-informative markers (AIMs) [20] genotyped by the GoldenGate BeadArray (Illumina) was extracted from an initial phase of the study where a subset of samples from the Guadalajara cohort was also analyzed [12].

To uncover evidence of population substructure, we conducted a multidimensional scaling (MDS) analysis as implemented in PLINK, using 225,635 SNPs that passed QC measures, in the discovery sample (Morelos cohort). Population stratification for the replication sample was evaluated using principal components produced from the principal component analysis (PCA), estimated using PLINK and the smartpca program in EIGENSOFT v3.0 package [21, 22], and ancestry estimates were included as confounding factors for correction of population stratification.

### 2.4. Imputation

IMPUTE 2.3.1 software [23] was used to impute the regions (1 Mb) that were associated with the FN and LS BMD traits. A regional imputation was performed with the 1000 Genomes Phase I integrated variant as a reference. To reanalyze the data with the imputed information, we performed a dosage analysis using PLINK software, which was adjusted for age, sex, BMI, and the first two principal components.

### 2.5. Conditional Analysis

To evaluate if the leading signals were independent, we performed a conditional analysis. For this analysis, we tested the association with BMD of L2–L4 and the FN using the leading SNP genotype as a covariate.

### 2.6. Selection of SNPs for Replication

In the replication analysis stage, we focused on the SNPs rs1432910 and rs2278391 (*SLIT3* gene), rs6904364 (*RMND1* gene), rs17081341 (*CCDC170* gene), rs849172 (*COA1* gene), rs11764843 (*HDAC9* gene), and rs17413103 (*SHFM1* gene), based on the following two main criteria: (1) these SNPs reached suggestive level of significance in the discovery analysis (*P* values <5 × 10^−5^) and (2) based on bioinformatics search criteria, these SNPs had a reported function in bone metabolism.

### 2.7. GEFOS Confirmation

We determined whether the SNPs that passed our threshold for replication for their association signals were in the GEFOS Consortium, the largest dataset for BMD in the field (release, 2012), which was downloaded from http://www.gefos.org/?q=content/data-release-2012. This release includes the summary data from the 2012 meta-analyses of GWA data. These files include *P* values for >2 million SNPs for association with femoral neck (in male, female, and all subjects) and lumbar spine BMD (for male, female, and pooled subjects), tested in 32,961 subjects from 17 studies. As this was a female GWAS, we used female-specific GEFOS data to perform a subsequent analysis.

### 2.8. Meta-Analysis

Results from the discovery and replication datasets were combined using an inverse variance fixed-effects meta-analysis that was implemented in METAL [24]. The contribution of each study to the meta-analysis was weighted by the standard error of the SNP association parameter (beta of the SNP effect). Cochran's *Q* test was used to analyze heterogeneity among study populations [25].

### 2.9. Comparison of Previously Known BMD Loci

In a complementary analysis, we evaluated the effect of loci known to be associated with BMD based on the GEFOS (~63 loci) [16] in the Mexican population. For this purpose, (1) we selected a SNP proxy based on LD > 0.8 using the online computer program SNAP Proxy Search (Broad Institute) to find the SNPs present in the Human CytoSNP-12 DNA v2.1 array, (2) the SNPs in linkage disequilibrium with the region detected in GEFOS and present in the CytoSNP-12 array were evaluated, and (3) a single SNP association was performed for each locus using the efficient mixed-model association expedited (EMMAX) to account for population stratification and hidden relatedness. The association tests were adjusted for potential confounders such as age, BMI, and ancestry estimates. To be considered statistically significant, a *P* value was calculated based on the number of evaluated SNPs (*P* = 0.0011, 0.05/43).

### 2.10. eQTL Analyses

To evaluate whether associated SNPs in the identified loci were involved in the regulation of messenger RNA levels via eQTLs, we queried publicly available genome-wide expression datasets using Sherlock online tool (http://sherlock.ucsf.edu/) [26] which integrates data of monocytes [27], and HaploReg [28] is a tool for exploring the effect of SNPs on expression from eQTL studies and integrates resources from the Genotype-Tissue Expression (GTEx) [29] and published eQTL studies.

### 2.11. Sample Size and Statistical Analysis

Based on results of previous meta-analyses of GWASs [16, 30–34] and previous studies in the Mexican population [7–13], we assumed that the effect size varies between 0.03 and 0.05 (beta values per standard deviation of BMD). Statistical power was calculated with Quanto 1.1 software [35], for a significance level of 5 × 10^−8^ and MAF of 5% in 831 postmenopausal women with a minimal power of 80% to detect differences in BMD, under an additive model. All data from the population in the study are shown as the mean ± SD (standard deviation) for the quantitative variables and as the absolute and relative frequencies for the qualitative variables. In the discovery sample, a single SNP association was performed with the efficient mixed-model association expedited (EMMAX) to account for population stratification and hidden relatedness. The association tests were adjusted for potential confounders such as age, BMI, and ancestry estimates. The Manhattan plots were generated using R 2.11.1.

In the replication sample, the Hardy-Weinberg equilibrium was tested for each SNP using the chi-square test. Linear regression analyses were used to estimate the effect of each SNP on BMD using an additive genetic model adjusted for age and body mass index (BMI). The analyses for BMD measured at the femoral neck and lumbar spine were performed separately. All statistical analyses were performed with the Statistical Package for Social Sciences software (SPSS 20.0; SPSS Inc., Chicago, IL, USA). Linkage disequilibrium (LD) and haplotype frequencies were estimated using Haploview 4.2 [36].

## 3. Results

### 3.1. Study Participants

The demographic and clinical characteristics of the participating women in the discovery and replication samples are presented in the supporting information (Table S1 available online at https://doi.org/10.1155/2017/5831020). In the discovery sample, the mean age was 62.24 ± 9.11 years and the mean BMI was 28.08 ± 4.76 kg/m^2^. The mean bone parameters were within normal ranges, which were expected given the age of the participants. In the replication sample, the mean age was 58.82 ± 8.08 years and the mean BMI was 29.17 ± 4.63 kg/m^2^. The mean bone parameters (FN and LS BMD) were within normal ranges. We observed significant differences between cohorts (*P* ≤ 0.05) in the following variables: age, BMI, FN BMD, number of children, years of menopause, tobacco use, and alcohol intake.

After the principal component analysis, the PC1 and PC2 values were plotted for the discovery sample and the replication sample (Figure S1), which supported that the population distribution in both cohorts of the study was consistent with the reported population distribution for admixed populations. Admixed populations are not ancestrally homogeneous but rather are populations with ancestry from more than one parental population [2, 3].

### 3.2. Discovery Sample: Femoral Neck (FN) BMD

The discovery sample consisted of the bone mineral density measurements of 411 postmenopausal women. The results from the GWAS for FN are shown in Figure S2A. None of the SNPs met the conventional criteria for genome-wide significance. Based on suggestive significance signals, we established a threshold of *P* < 5.0 × 10^−5^ for the replication analysis. Details of the SNPs with evidence of association with the FN BMD based on the established threshold are shown in Table S2. Twelve SNPs in the FN BMD GWAS exceeded our significance threshold criteria.

**Figure 1:**
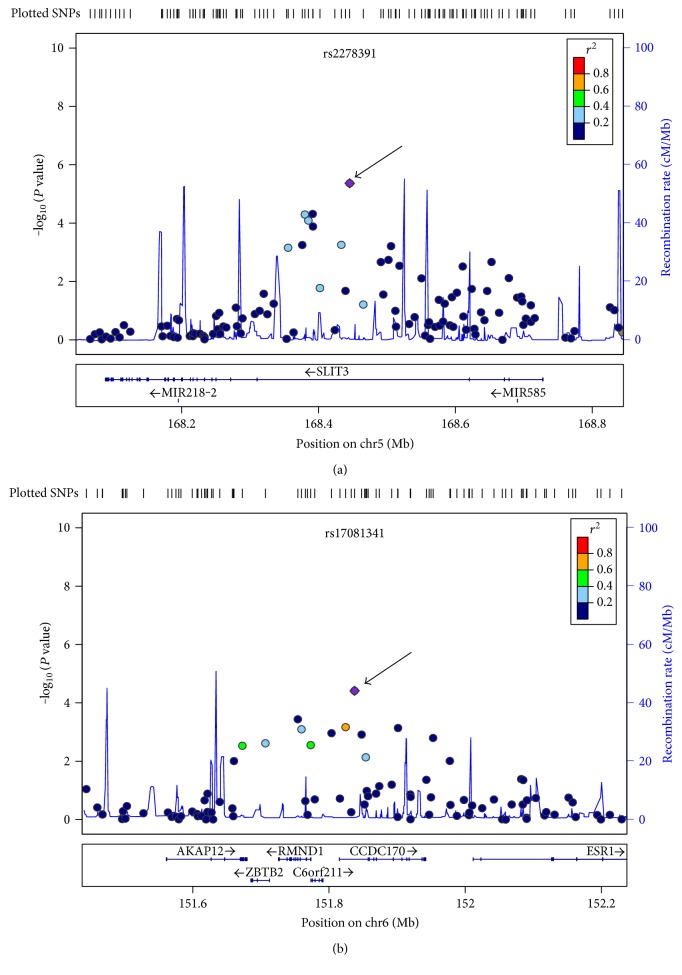
Evidence of association of BMD in the femoral neck with the *SLIT3* and *CCDC170* chromosomal regions. The *x*-axis is the physical position on the chromosome (Mb), and the *y*-axis denotes the association test result as the −log (*P* value). SNPs are color-coded according to the linkage disequilibrium (LD) with the lead SNP (purple diamond) indicated by the arrow. (a) The SNP rs2278391 in the *SLIT3* region; (b) the SNP rs17081341 in the RMND1/*CCDC170* region. The regional association plots in the Morelos cohort were generated using LocusZoom and only show genotyped SNPs.

The most significantly associated intergenic SNP (rs2573223) was on chromosome 2, ~12 kb to the *PRSS56* gene (*P* = 1.81 × 10^−6^), followed by two intronic SNPs on the *SLIT3* gene on chromosome 5 (rs2278391, *P* = 4.31 × 10^−6^ and rs1432910, *P* = 4.84 × 10^−5^) (Figure 1(a)). Interestingly, the association extended to SNPs with similar minor allele frequency (rs752498, MAF = 0.44, *P* = 5.05 × 10^−5^ and rs6555841, MAF = 0.46, *P* = 8.17 × 10^−5^), and the LD patterns among them revealed that they could be grouped into two clusters on the *SLIT3* gene (Figure S3A).

The second most significant result was obtained with an intronic SNP on chromosome 6 in the *CCDC170* gene before named *C6orf97* (rs17081341, *P* = 3.86 × 10^−5^) (Figure 1(b)). The association extended to a nearby intronic SNP, rs6904364 (*P* = 0.0003; MAF = 0.62), in the *RMND1* gene. These SNPs are located in two linkage disequilibrium blocks; rs6904364 is located within block 1 (24 kb), such that rs17081341 (block 3–13 kb) is in a moderately high LD with rs6904364 [*D*′ = 0.76] (Figure S3B).

### 3.3. Discovery Sample: Lumbar Spine (LS) BMD

**Figure 2:**
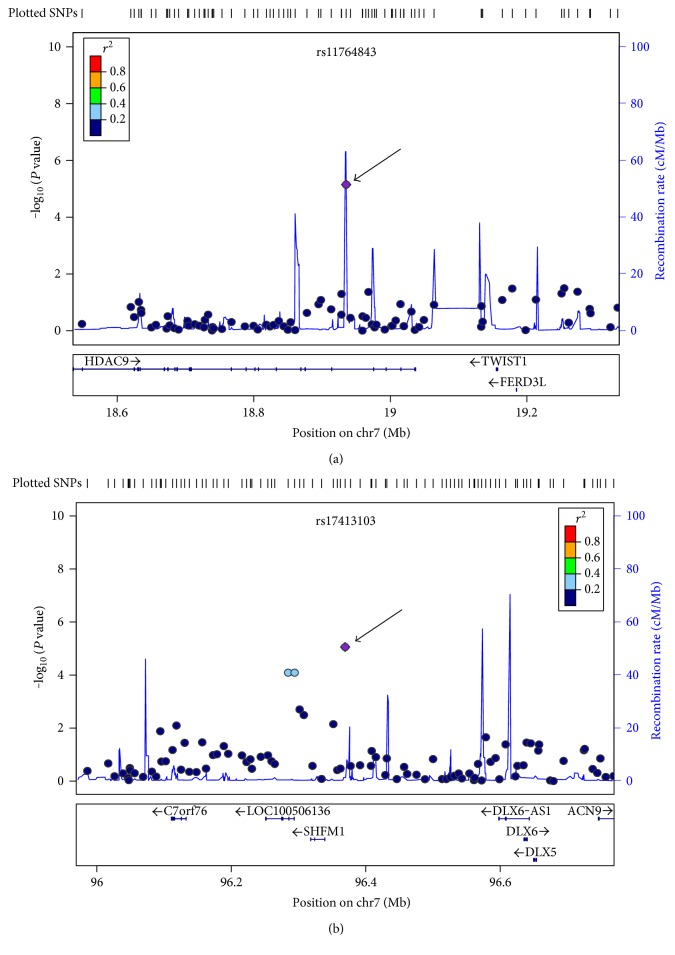
Regional association plots for the lumbar spine BMD in the *HDAC9* and *SHFM1* chromosomal regions. The *x-*axis is the physical position on the chromosome (Mb), and the *y*-axis denotes the association test result as the −log (*P* value). SNPs are color-coded according to the linkage disequilibrium (LD) with the lead SNP (purple diamond) indicated by the arrow. (a) The SNP rs11764843 in the *HDAC9* region; (b) the SNP rs17413103 in the *SHFM1* region. The regional association plots were generated using LocusZoom and only show genotyped SNPs.

The results from the (LS) analysis are shown in Figure S2B. Similar to the SNPs in the FN BMD analysis, none of the SNPs in the lumbar spine exceeded the conventional criteria for genome-wide significance; therefore, we also applied our threshold of *P* < 5.0 × 10^−5^ to the lumbar spine analysis. Eleven SNPs in the LS BMD GWAS meet our arbitrary threshold criteria for suggestive genome-wide significance (Table S3). The most significant results were observed for the intronic SNP rs10446738 on chromosome 4 (*P* = 2.04 × 10^−6^), intronic rs7221458 on chromosome 17 in the *LINC01563* gene, and two intronic SNPs on chromosome 7: one in the *HDAC9* gene (rs11764843; *P* = 7.12 × 10^−6^) (Figure 2(a)) and the other in the *SHFM1* gene (rs17413103; *P* = 8.76 × 10^−6^) (Figure 2(b). The two associated SNPs on chromosome 7 were located within a linkage disequilibrium region, with high LD (*D*′ = 0.85). However, the SNP rs11764843 was mapped to a region with a high recombination rate, which is the same region that shapes haplotype blocks (Figures 2(a) and S4A), and the second SNP rs17413103 has a moderate recombination rate between blocks 1 and 2 (Figures 2(b) and S4B).

We observe another two associated SNPs near rs17413103, rs10261558, and rs12537768 (*P* = 8.13 × 10^−5^, MAF = 0.22 and *P* = 8.13 × 10^−5^, MAF = 0.22, resp.), which were both located within a linkage disequilibrium block with high LD (*D*′ = 0.90) (Figure S4B). Furthermore, an imputation analysis of the associated region was conducted to obtain additional information on the SNPs located in the regions associated with the FN and LS BMD phenotypes. However, none of the imputed SNPs showed *P* values of greater significance than the genotyped SNPs. Additionally, there was no sufficient evidence supporting their role in disease pathogenesis that would warrant further replication. Therefore, only the LocusZoom plots are shown for genotyped SNPs.

### 3.4. Conditional Analysis

A conditional analysis was performed to determine whether the association detected at the *RMND1*/*CCDC170* locus might be the result of multiple independent genetic factors in this region. In the rs6904364-rs17081341 region, because rs6904364 is physically closer to rs17081341 (distance: 49 kb), the conditional analysis was performed as follows: rs6904364 was conditional on rs17081341 under a fixed-effects model, which resulted in a *P* = 0.0031 for the FN BMD in the stage I discovery sample (data not shown). Considering that the region evaluated contained 119 SNPs, the alpha value for this conditional analysis was *P* = 0.00042. These results indicate that rs6904364 and rs17081341 represent the same signal at this chromosomal locus.

For *HDAC9*, which includes the SNPs rs11764843 and rs17413103 in the *SHFM1* region, the conditional analysis was performed as follows: rs11764843 was conditional on rs17413103 under a fixed-effects model, which resulted in a *P* = 2.62 × 10^−5^ for the lumbar spine. These results suggest that rs11764843 and rs17413103 represent distinct signals. Further studies in other Mexican populations are required to explain the significance of these association signals.

### 3.5. Replication Analysis, GEFOS Confirmation, and Meta-Analysis

In the replication analysis, seven SNPs were examined in an independent population of postmenopausal women from Guadalajara city, Mexico (Table S1). Under an additive model, a linear regression analysis adjusted for age and BMI, and the first two principal components, the results showed that only two SNPs were associated with the FN BMD: rs6904364 allele C [*β* = 0.027 (95% CI 0.002; 0.053), *P* = 0.032] and rs17081341 allele G [*β* = 0.041 (95% CI 0.008; 0.075), *P* = 0.015] on the *RMND1* gene (Table 1).

Using the GEFOS dataset (release, 2012) for in silico validation, the SNPs of the *SLIT3* and *HDAC* and those near to *SHFM1* genes did not achieve signals of association with BMD phenotypes. However, the consortium reported *P* values after a meta-analysis in FN BMD of 0.0082 for the rs6904364 located in the *RMND1* gene, of 0.0548 for rs170811341 in the *CCDC170* gene, and of 0.0497 for rs849172 in the *COA1* gene (Table 1). In the meta-analysis of both the discovery and replication samples, evidence for the association of FN BMD with the *RMND1* SNP became significant; the *P* value in the meta-analysis for rs6904364 was 2.77 × 10^−4^, and for the association of the *CCDC170* SNP, the *P* value for rs17081341 was 1.62 × 10^−5^. For the two SNPs, the meta-analysis did not substantially improve the evidence of the association observed in the discovery sample. Heterogeneity in effect sizes between the discovery and the replication samples was not significant for all SNPs (Table 1).

### 3.6. Frequency Comparison with Other Populations

**Figure 3:**
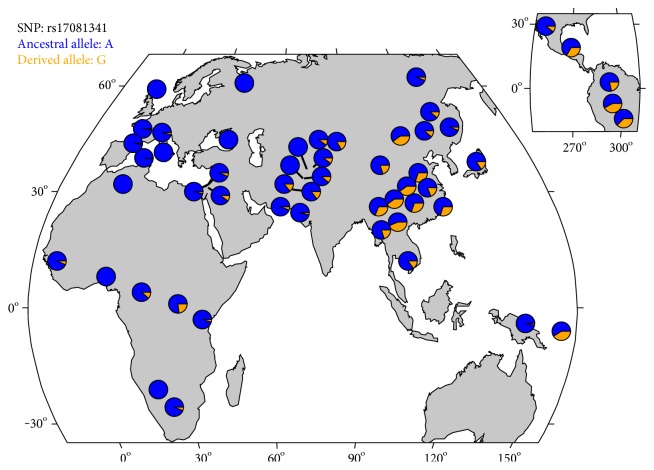
Allele frequency distribution of the SNP rs17081341 in the Human Genome Diversity Panel dataset.

Table 2 shows the minor allele frequency (MAF) distribution between Mexican-Mestizos (MX), Utah residents with northern and western European ancestry (CEU), Los Angeles residents with Mexican ancestry (MXL), and Amerindian (AMR) populations. The rs17081341 “G” allele on the *CCDC170* gene, which was reported to be not associated with the FN BMD in the GEFOS dataset, was more frequent in the Mexican-Mestizo populations (0.17) than in the European populations (0.03), and the frequency in Mexican-Mestizo populations was very similar to the frequency observed in the Los Angeles population of individuals with Mexican ancestry (0.16). Interestingly, Amerindians had a higher frequency (0.24) of the rs17081341 “G” allele on the *CCDC170* gene. With regard to the remaining SNPs, the frequencies did not vary significantly among the compared populations. Using the data generated by the 1000 Genomes Project, we observed that the rs17081341 “G” allele on the *CCDC170* gene was rare or absent in the samples from Europe and had intermediate frequency (7–12%) in the South Asian and African samples and a high frequency (20%) in the samples from the Asian populations, whereas frequencies up to 22% were observed in the samples from the Amerindian populations (Figure 3).

### 3.7. Comparison of Previously Known BMD Loci

Of the ~63 loci identified by Estrada et al. [16], 43 proxies were available for analysis in our discovery sample results (Table S4). Among them, six were significant for the FN BMD and five were significant for the LS BMD (*P* = 0.001). The SNP rs3779381 was a proxy for the SNP rs3801387 on the *WNT16* gene in the GEFOS dataset and showed a trend towards an association with the FN and LS BMD (*P* = 0.007 and 0.005, resp.), which was above the multiple test significance threshold that was estimated at 0.001 (0.05/43). After correcting for multiple testing, the association of FN BMD with the rs4870044 SNP, which was a proxy for the rs4869742 SNP in the GEFOS dataset and rs7128738 on the SOX6 gene, remained significant (*P* = 0.0007 and 0.001, resp.). For the LS BMD, only rs11887431, which was a proxy for the rs7584262 SNP on the PKDCC gene in the GEFOS dataset, remained significant (*P* = 0.0006, Table 3).

### 3.8. eQTLs

We perform a primary analysis, to determine if any of the associated variants with femoral neck and lumbar spine BMD in the discovery sample has eQTL effects using Sherlock online tool [26] and HaploReg [27]. In the Sherlock online tool, no significant genes were discovered in the analysis. We found evidence for two SNPs in the HaploReg, the rs6904364 at the *RMND1* locus influencing the expression of RMND1 in muscle-skeleton and the rs849172 at the *COA1* locus influencing expression of COA1 in muscle-skeleton and and BLVRA expression in whole blood [29]. These tissues have no functional relevance to BMD and bone metabolism, and thus, additional analyses in more relevant cell types such as osteoblasts, osteoclasts, and monocytes as osteoclast precursors will be to identify the target transcripts regulated by these GWAS signals. In the future, extensive analyses of eQTL gene expression in BLUEPRINT data [37] and the Biobank-based Integrative Omics Study (BIOS) [38, 39] will improve the candidate gene search involved in the variation of BMD, in Mexican Population.

## 4. Discussion

Here, we have described the results of a pilot genome-wide association study and replication analysis; these results are suggestive and they are hypothesis generating and highlighting a potential role of the estrogen receptor in bone metabolism. Also, this study represents the first high-density large-scale GWAS conducted in a Mexican-Mestizo population that has targeted the FN and LS BMD. Mexican-Mestizos are an admixed population of Native Americans (51%), Europeans (45.4%), and a small percentage of African ancestry (3.7%) [2, 3]. As expected, given the European ancestry in the Mexican-Mestizo population, our study replicated associations previously reported in European populations and the suggestive findings in this study may be of value for future studies with larger sample sizes in other Mexican populations.

The major advantage of genome-wide association studies is the ability to identify novel genes without prior knowledge of the function of the genes. In this regard, studies have demonstrated that SLIT3 promotes the direct migration of monocytes triggered by chemoattractants in vitro [40]. It is important to note that circulating monocytes are key cells that participate in osteoclastogenesis by acting as osteoclast precursors [41] and produce a wide variety of factors that are involved in bone metabolism by regulating osteoclastic differentiation. Using the replication sample and GEFOS dataset, we did not confirm association signals for these SNPs, suggesting that the loci identified in this study may contribute to BMD variation only at a specific population level.

Among the loci associated with the LS BMD, only *HDAC9* could play an important role in bone metabolism. A recent study suggests that *HDAC9* (histone deacetylase 9) controls bone turnover by suppressing osteoclast differentiation. *HDAC9*-knockout mice exhibit elevated bone resorption and lower bone mass [42]. These findings identify *HDAC9* as a novel candidate gene and an important and physiologically relevant modulator of bone remodeling and skeletal homeostasis [43]. This hypothesis needs to be tested in future studies with larger sample sizes.

Although there are no published data concerning the function of the *SHFM1* gene in bone metabolism, the rs17413103 SNP in this gene, which was associated with FN BMD in our discovery sample, is located ~200 kb upstream of the rs10429035 SNP on the *C7orf76* gene. This region was recently reported to be associated with hip BMD [44]. However, rs10429035 is not included in the Human CytoSNP-12 DNA v2.1 array used in this study. To determine if rs17413103 is associated with the BMD phenotypes in our population, additional studies (e.g., densely imputed data) are required in this region and with other Mexican-Mestizo populations. However, discovery finding of these loci did not replicate in the Mexican population, or in GEFOS. Additional studies are necessary to clarify the role of these loci in the variation of bone mineral density, in other Mexican populations.

There were at least two linkage disequilibrium blocks that were associated with femoral neck in our population, rs6904364 in the *RMND1* gene and rs1708134 located within the *CCDC170* gene. The *CCDC170* gene encodes a protein with an unknown function and is located contiguous to the *ESR1* gene. It is possible that the associations in this region between the *CCDC170* gene and BMD may reflect the existence of additional genetic factors that affect variations in BMD in the Mexican-Mestizo population and the presence of important regulatory elements relevant to the *ESR1* gene. Nevertheless, this association pattern is in agreement with the results of a previous genome-wide association study in an Icelandic sample [31]. These data and the results of previous studies suggest that high-density SNP genotyping is necessary in larger samples and other Mexican-Mestizo populations to further analyze the complex genomic architecture of variations in BMD and osteoporosis in admixed populations.

Conversely, the meta-analysis of the discovery and replication samples resulted in evidence of an association of rs17081341 and rs6904364 with BMD, which suggests that the association between these SNPs and BMD could be specific for the Mexican-Mestizo population or that this locus may be the same signal as reported by Styrkarsdottir et al. [31]. To date, the loci reported to be associated with osteoporosis have small effects on variations in BMD [16, 30–34, 45]. Additionally, in the Mexican population, individuals from different geographic regions have significantly different ancestries [46, 47].

The rs17081341 “G” allele of the *CCDC170* gene was observed more frequently in Mexican-Mestizo individuals, individuals from Los Angeles of Mexican ancestry, and Amerindian populations (0.17, 0.16, and 0.24, resp.) than in Europeans (0.03) (Table 2). The low frequency of this allele in Europeans suggests that this variant is highly prevalent in a specific subset of humans, such as Native Americans and Native American-derived populations (i.e., Mexican-Mestizo population); however, this variant may also be present in some non-Amerindian populations such as Asians. In addition, it has been widely documented that some genetic variants are present at different frequencies across populations. Therefore, some variants may have a higher frequency in one population (Mexican-Mestizo population) but a rare or low frequency in another (European population). As a result, some variants may be overlooked in large studies of European and Asian populations. This could explain why rs6904364 on the *RMND1* gene and rs17081341 on the *CCDC170* gene have not been associated with BMD in large studies in other populations. Using the GEFOS dataset, we confirmed in silico association signals for the rs17081341 and rs6904364 SNPs, suggesting that the loci identified in this study may also contribute to BMD variation across the different populations. The GEFOS is the largest dataset for an osteoporosis GWAS meta-analysis [16]. Therefore, the GEFOS data used in our in silico replication here are strong and are/could be used as benchmark data for SNP association signals for osteoporosis.

We also compared our results with the approximately 63 loci found to be associated with BMD in the GEFOS dataset [16]. Interestingly, SNPs in the *SOX6* and *PKDCC* genes showed site-specific effects on BMD (femoral neck and lumbar spine). To our knowledge, these genes have not been previously associated with variations in BMD or osteoporosis in a Mexican-Mestizo population. However, these genes have previously been reported to affect BMD in Asian and European populations, which suggest that both shared and unique genetic backgrounds associated with BMD are present across different ethnic groups. Additional studies are required to understand the role of *RMND1*, *CCDC170*, *SOX6*, and *PKDCC* in variations in BMD in the Mexican population. These results could also serve as a reference and may be informative for future studies in Mexican-Mestizo populations.

Our study has some limitations. First, the failure to replicate the previously reported associations between SNPs and BMD in European populations may be due to the small sample size and reduced statistical power of the study. These factors might also have prevented us from discovering additional (not only low-frequency) variants [48]. Although the sample size (*n* = 831) was large enough to identify the effect of several variants on BMD, it is not comparable with the sample size of other GWASs conducted in European and Asian populations that analyzed thousands of samples. Based on our observed MAF (0.11–0.49) and effect size (0.03–0.076, in standard deviations of BMD), we would need >3500 samples to achieve 80% statistical power to identify genome-wide significant associations with BMD in our population. The statistical power of the present study to find a significant association with the SNPs rs6904364 and rs17081341 was 21% and 43%, respectively. Second, we cannot rule out the possibility that unidentified genetic variants associated with bone mineral density have been missed due to incomplete coverage of the genome by the genotyping platform used in this study (i.e., Illumina HumanCytoSNP), which was not designed to be used in GWAS; future fine mapping analyses on candidate genes will address this question. Third, this analysis is significantly weakened by the lack of genome-wide imputation. Fourth, these results could reflect the complex genetic architecture of BMD variation in the Mexican population, which is determined not only by genetic factors but also by environmental factors. Populations from the same ethnic origin but different geographic regions (such as the populations used in this study) have varying exposures to environmental factors, such as alcohol consumption, smoking, diet, and exercise, which may play an important role in variations in BMD in Mexican-Mestizo and other populations [47, 49]. However, it is difficult to accurately assess the contribution that these factors have on bone mineral density variation.

Finally, due to the problems associated with data availability and the difficulty of standardizing questionnaires across studies, we did not evaluate the effect of potential confounders such as years since menopause and hormone replacement therapy (HRT) can influence genetic associations with BMD. Nonetheless, despite these limitations, we have identified a previously reported variant associated with variations in BMD. In addition, the current study and recent publications [50–54] demonstrated the value of studying diverse populations and/or specific population variability and attempt to replicate the results of GWAS, and other genetic studies in ethnically different populations, such as Amerindian descendants, are necessary and could provide valuable contributions to understanding variations in BMD.

In conclusion, this study represents the first high-density large-scale GWAS for variations in BMD carried out in Amerindians (Mexican-Mestizo population). Our results independently confirm previously identified loci implicated in BMD, and further studies are necessary to confirm the suggestive findings.

## Supplementary Material

Table S1 Demographic characteristics and BMD of the discovery and replication samples. Table S2 Top SNPs associated with FN-BMD in the discovery sample (P < 5 x 10-5). Table S3 Top SNPs associated with LS-BMD in the discovery sample (P < 5 x 10-5). Table S4 Proxy SNPs for the GEFOS included in the discovery sample. Figure S1 Principal component analysis calculated for all the Postmenopausal women included in the study. The colors in the legend denote the ethnic group: EUR, AFR, NAT, MOR and GUAD. Postmenopausal women of Discovery sample (MOR), PC1 and PC2 were calculated using 60, 793 AIMs. Postmenopausal women of replication sample (GUAD), PC1 and PC2 were calculated using 96 AIMs with MAF > 5% and missing call rate < 5%. Figure S2 Manhattan plots of the genome-wide association results of the discovery sample. Figure S3 Linkage Disequilibrium (LD) plots with the D' values for the SLIT3 and CCDC170 genes. Figure S4 Linkage Disequilibrium (LD) plots with the D' values for the HDAC9 and SHFM1 genes.

## Figures and Tables

**Table 1:** Associated SNPs with femoral neck and lumbar spine BMD in discovery and replication samples.

SNP	Physical position	CHR	Gene	A1/A2^a^	Discovery BMD^b^			Replication BMD^c^			Meta-analysis	P-Het^d^	GEFOS look-up^e^		
					MAF	*β*	*P*	MAF	*β*	*P*	*P*		MAF	*β*	*P*
rs1432910	168,391,557	5	*SLIT3*	T/C	0.48	−0.031	4.84 × 10^−5^	0.48	−0.015	0.231	—	0.264	0.49	−0.012	0.598
rs2278391	168,445,608	5	*SLIT3*	A/G	0.27	0.038	4.31 × 10^−6^	0.28	0.003	0.798	—	0.795	0.21	0.001	0.747
**rs6904364**	**151,754,273**	**6**	***RMND1***	**C/T**	**0.38**	**0.028**	**3.63 × 10** ^**−4**^	**0.38**	**0.027**	**0.032**	**2.77 × 10** ^**−4**^	0.993	**0.32**	**—**	**0.008**
**rs17081341**	**151,837,570**	**6**	***CCDC170***	**G/A**	**0.17**	**0.041**	**3.86 × 10** ^**−5**^	**0.13**	**0.041**	**0.015**	**1.62 × 10** ^**−5**^	0.999	**0.04**	**0.0001**	**0.054**
rs849172	43,765,667	7	*COA1*	T/C	0.44	0.033	1.79 × 10^−5^	0.49	−0.012	0.362	—	0.180	0.46	0.008	0.049
rs11764843	18,935,310	7	*HDAC9*	A/C	0.49	−0.045	7.12 × 10^−6^	0.48	−0.010	0.541	—	0.584	0.42	−0.008	0.521
rs17413103	96,369,421	7	*SHFM1*	A/C	0.11	0.076	8.76 × 10^−6^	0.09	−0.003	0.918	—	0.471	0.08	0.019	0.105

MAF: minor allele frequency; CHR: chromosome; *P* value for additive model, adjusted for age, body mass index, and principal components. — means information not available for this SNP. ^a^A1 represents the minor allele. ^b^Mexican-Mestizo women from Central México (Morelos state), *n* = 411. ^c^Mexican-Mestizo women from Western Mexico (Guadalajara City), *n* = 420. ^d^P-Het, P-heterogeneity for meta-analysis. ^e^GEFOS dataset release 2012; beta values obtained from GEFOS data release 2015.

**Table 2:** Allele frequency of SNPs associated with BMD phenotypes in the discovery sample and comparison with other populations.

			Minor allele frequency			
SNP	CHR	*Gene*	CEU^a^	MXL^a^	MX^b^	AMR^c^
rs1432910	5	*SLIT3*	0.56	0.44	0.48	0.58
rs2278391	5	*SLIT3*	0.16	0.24	0.27	0.39
rs6904364	6	*RMND1*	0.37	0.41	0.38	0.36
rs17081341	6	*CCDC170*	0.03	0.16	0.17	0.24
rs11764843	7	*HDAC9*	0.65	0.48	0.49	—
rs17413103	7	*SHFM1*	0.09	0.09	0.11	0.11

— means information not available for this SNP; CEU: Utah residents with Northern and Western European ancestry; MXL: Mexican Ancestry in Los Angeles, California. ^a^Data obtained from 1000 Genomes dataset. ^b^MX: Mexican-Mestizo population from Central México (Morelos state). ^c^AMR: Amerindian population of Nahua, Totonac, and Zapotec. Data were obtained from Consortium for the Study of Genomic Diversity of the Indigenous Populations.

**Table 3:** Comparison of SNP effect sizes for BMD between GEFOS genome-wide meta-analysis and this study.

GEFOS genome-wide meta-analysis^a^					This study		
SNP	Locus	Gene	Beta	*P* value	Proxy SNP	Beta	*P* value
*Femoral neck BMD*							
rs1026364	3q13.2	*KIAA2018*	0.03	8.86 × 10^−7^	rs9813630	0.018	0.046
**rs4869742**	**6q25.1**	***CCDC170***	**−0.05**	**3.14 × 10** ^**−13**^	**rs4870044**	**−0.025**	**0.0007**
rs3801387	7q31.31	*WNT16*	−0.08	2.78 × 10^−33^	rs3779381	−0.025	0.007
rs13245690	7q31.31	*CPED1*	0.02	8.20 × 10^−4^	rs13245690	0.021	0.026
**rs7108738**	**11p15.2**	***SOX6***	**−0.08**	**3.52 × 10** ^**−26**^	**rs7128738**	**−0.029**	**0.001**
rs227584	17q21.31	*C17orf53*	−0.06	3.15 × 10^−18^	rs227584	−0.016	0.037
*Lumbar spine BMD*							
**rs7584262**	**2p21**	***PKDCC***	**0.01**	**0.05**	**rs11887431**	**0.057**	**0.0006**
rs344081	3q25.31	*LEKR1*	0.05	1.49 × 10^−8^	rs344081	0.043	0.032
rs3801387	7q31.31	*WNT16*	−0.11	6.38 × 10^−51^	rs3779381	−0.036	0.005
rs13245690	7q31.31	*CPED1*	0.05	1.65 × 10^−11^	rs13245690	0.031	0.020
rs7108738	11p15.2	*SOX6*	−0.03	5.76 × 10^−5^	rs7128738	−0.030	0.021

^a^Data of effect sizes for the SNP and BMD site (stage 1 + stage 2) were obtained from Supplemental Table S5, Estrada et al. [16]. Bold indicates *P* values > 0.001 (*P* = 0.05/43).
