# Supplementary material for: A Pilot Genome-Wide Association Study in Postmenopausal Mexican-Mestizo Women Implicates the RMND1/CCDC170 Locus Is Associated with Bone Mineral Density
Source: Int J Genomics. 2017 Aug 3;2017:5831020. doi: 10.1155/2017/5831020 (PMC5559934; doi:10.1155/2017/5831020)
Supplement: Supplementary file 1 — Table S1 Demographic characteristics and BMD of the discovery and replication samples. Table S2 Top SNPs associated with FN-BMD in the discovery sample (P < 5 x 10^-5^). Table S3 Top SNPs associated with LS-BMD in the discovery sample (P < 5 x 10^-5^). Table S4 Proxy SNPs for the GEFOS included in the discovery sample. Figure S1 Principal component analysis calculated for all the Postmenopausal women included in the study. The colors in the legend denote the ethnic group: EUR, AFR, NAT, MOR and GUAD. Postmenopausal women of Discovery sample (MOR), PC1 and PC2 were calculated using 60, 793 AIMs. Postmenopausal women of replication sample (GUAD), PC1 and PC2 were calculated using 96 AIMs with MAF > 5% and missing call rate < 5%. Figure S2 Manhattan plots of the genome-wide association results of the discovery sample. Figure S3 Linkage Disequilibrium (LD) plots with the D' values for the SLIT3 and CCDC170 genes. Figure S4 Linkage Disequilibrium (LD) plots with the D' values for the HDAC9 and SHFM1 genes. [file 5831020.f1.docx]

**Supporting Information**

*Title:* Genome-wide association study in postmenopausal Mexican-Mestizo women implicates *RMND1/CCDC170* loci as associated with bone mineral density.

Marisela Villalobos-Comparán^1^, Rogelio F. Jiménez-Ortega^2^, Karol Estrada^3^, Alma Y. Parra-Torres^2^, Anahí González-Mercado^4^, Nelly Patiño^5^, Manuel Castillejos-López^6^, Manuel Quiterio^7^, Juan Carlos Fernandez-López^1^, Bertha Ibarra^4^, Sandra Romero-Hidalgo^1^, Jorge Salmerón^7, 8^ and Rafael Velázquez-Cruz^2^.

***Author affiliations:***

**^1^**Consorcio de Genómica Computacional, Instituto Nacional de Medicina Genómica, Mexico City, Mexico.

**^2^**Laboratorio de Genómica del Metabolismo Óseo, Instituto Nacional de Medicina Genómica, Mexico City, Mexico.

^3^Statistical Genetics, Biogen, Cambridge, MA, USA.

^4^Doctorado en Genética Humana, Centro Universitario en Ciencias de la Salud, Universidad de Guadalajara and División de Genética, Centro de Investigación Biomédica de Occidente, IMSS, Guadalajara, Jalisco, México.

^5^Subdirección de Desarrollo de Aplicaciones Clínicas, Instituto Nacional de Medicina Genómica, Mexico City, Mexico

^6^Unidad de Vigilancia Epidemiológica Hospitalaria, Instituto Nacional de Enfermedades Respiratorias, Mexico City, Mexico.

^7^Centro de Investigación en Salud Poblacional, Instituto Nacional de Salud Pública, Cuernavaca, Morelos, Mexico

^8^Unidad de Investigación Epidemiológica y en Servicios de Salud, Instituto Mexicano del Seguro Social, Cuernavaca, Morelos, Mexico

**Corresponding author:** Rafael Velázquez-Cruz PhD. Laboratorio de Genómica del Metabolismo Óseo, Instituto Nacional de Medicina Genómica. Periférico Sur No. 4809, Col. Arenal Tepepan, Delegación Tlalpan. México, D.F. C.P. 14610,

Phone. +52 (55) 5350-1900

Fax 5350-1999.

E-mail: rvelazquez@inmegen.gob.mx

**Supplementary Tables and Figures**

**Tables:**

**Table S1 Demographic characteristics and BMD of the discovery and replication samples.**

|  | **Morelos** | **Guadalajara** | ***P-value*** |
| --- | --- | --- | --- |
| **Variable** | **Mean (SD)** | **Mean (SD)** |  |
| Age (Yr) | 62.2 (9.1) | 58.8 (8.0) | <0.0001 |
| Height (cm) | 152.9 (5.7) | 154.2 (5.5) | 0.0007 |
| Weight (Kg) | 65.7 (11.6) | 69.6 (11.1) | <0.0001 |
| BMI^a^ (Kg/m^2^) | 28.0 (4.7) | 29.1 (4.6) | 0.0007 |
| BMD^b^ femoral neck (g/cm^2^) | 0.8 (0.1) | 0.8 (0.1) | 0.0473 |
| T-Score femoral neck | -1.2 (0.9) | -1.2 (0.7) | 0. 6925 |
| BMD^b^ lumbar spine (g/cm^2^) | 0.9 (0.1) | 0.9 (0.1) | 0.1597 |
| T-Score lumbar spine | -1.0 (1.2) | -1. 6 (1.2) | 0.5669 |
| Age of Menarche | 12.9 (1.5) | 13.0 (1.5) | 0.2783 |
| Number of children | 3.0 (2.1) | 6.1 (3.7) | <0.0001 |
| Duration of breastfeeding (months) | 15.9 (19.8) | -- | -- |
| Years since menopause | 13.3 (11.1) | 12.6 (9.2) | < 0.0001 |
| Estrogen replacement therapy^c^ | 110 (26.0) | -- | -- |
| Tobacco use^c^ | 137 (34.3) | 48 (10.7) | < 0.0001 |
| Alcohol intake^c^ | 45 (11.2) | 3 (0.6) | < 0.0001 |
| Carbonated beverage consumption^c^ | 315 (78.7) | -- | -- |

“--“ means information not available

*n*=411 Postmenopausal women (Morelos).

*n*=420 Postmenopausal women (Guadalajara).

*^a^BMI* = *Body Mass Index*;

*^b^BMD*=*Bone Mineral Density*

^c^n (%); n, number of women

SD, standard deviation.

**Table S2 Top SNPs associated with FN-BMD in the discovery sample (*P* <5 x 10^-5^).**

| **SNP ID** | **Chr** | **Position^a^** | ***Gene*** | **A1^b^** | **A2** | **MAF^c^** | **β** | **SE** | ***P-value*** |
| --- | --- | --- | --- | --- | --- | --- | --- | --- | --- |
| rs2573223 | 2 | 233372850 | *near PRSS56* | G | A | 0.16 | 0.050 | 0.010 | 1.81 x 10^-6^ |
| rs2651562 | 4 | 101506795 | *--* | G | A | 0.11 | 0.052 | 0.012 | 1.60 x 10^-5^ |
| rs1432910 | 5 | 168391557 | *SLIT3* | T | C | 0.48 | -0.031 | 0.007 | 4.84 x 10^-5^ |
| rs2278391 | 5 | 168445608 | *SLIT3* | A | G | 0.27 | 0.038 | 0.008 | 4.31 x 10^-6^ |
| rs17081341 | 6 | 151837570 | *CCDC170* | G | A | 0.17 | 0.041 | 0.010 | 3.86 x 10^-5^ |
| rs849172 | 7 | 43765667 | *COA1* | T | C | 0.44 | 0.033 | 0.008 | 1.79 x 10^-5^ |
| rs673982 | 7 | 51846147 | *--* | A | G | 0.22 | 0.037 | 0.008 | 1.44 x 10^-5^ |
| rs611927 | 11 | 56815413 | *--* | A | C | 0.40 | 0.034 | 0.008 | 1.11 x 10^-5^ |
| rs612948 | 11 | 56930712 | *--* | A | G | 0.40 | 0.032 | 0.007 | 1.63 x 10^-5^ |
| rs8003062 | 14 | 96551440 | *C14orf132* | A | G | 0.45 | -0.032 | 0.008 | 4.44 x 10^-5^ |
| rs6029712 | 20 | 35465810 | *SOGA1* | G | A | 0.18 | -0.043 | 0.010 | 1.23 x 10^-5^ |
| rs9612051 | 22 | 43750704 | *near SCUBE1* | A | G | 0.12 | 0.049 | 0.012 | 3.04 x 10^-5^ |

“--“ means information not available.

^a^Chromosomal position (Mb) based on human genome build 19, dbSNP build 37.

^b^allele minor.

^c^Minor allele frequency (MAF) estimated from the discovery samples.

**Table S3 Top SNPs associated with LS-BMD in the discovery sample (*P* <5 x 10^-5^).**

| **SNP ID** | **Chr** | **Position^a^** | ***Gene*** | **A1^b^** | **A2** | **MAF^c^** | **β** | **SE** | ***P-value*** |
| --- | --- | --- | --- | --- | --- | --- | --- | --- | --- |
| rs12502642 | 4 | 2714023 | *FAM193A* | A | G | 0.32 | -0.047 | 0.011 | 4.34 x 10^-5^ |
| rs1216405 | 4 | 129527656 | *--* | A | G | 0.38 | -0.048 | 0.011 | 1.30 x 10^-5^ |
| rs10446738 | 4 | 129540702 | *--* | A | G | 0.20 | -0.066 | 0.014 | 2.04 x 10^-6^ |
| rs11764843 | 7 | 18935310 | *HDAC9* | A | G | 0.49 | -0.045 | 0.010 | 7.12 x 10^-6^ |
| rs7807686 | 7 | 83295674 | *near SEMA3E* | A | C | 0.24 | -0.055 | 0.013 | 1.78 x 10^-5^ |
| rs17413103 | 7 | 96369421 | *near SHFM1* | A | C | 0.11 | 0.076 | 0.017 | 8.76 x 10^-6^ |
| rs1946039 | 11 | 131052399 | *LOC105369578* | A | G | 0.48 | 0.045 | 0.011 | 3.30 x 10^-5^ |
| rs2589315 | 13 | 43743464 | *near ENOX1* | G | A | 0.32 | -0.049 | 0.012 | 2.99 x 10^-5^ |
| rs7221458 | 17 | 20980848 | *LINC01563* | A | G | 0.43 | 0.051 | 0.011 | 7.07 x 10^-6^ |
| rs12977265 | 19 | 13472019 | *CACNA1A* | G | A | 0.06 | -0.098 | 0.023 | 2.08 x 10^-5^ |
| rs2242854 | 21 | 16988490 | *--* | A | G | 0.11 | -0.073 | 0.017 | 3.17 x 10^-5^ |

“--“ means information not available.

^a^Chromosomal position (Mb) based on human genome build 19, dbSNP build 37.

^b^allele minor.

^c^Minor allele frequency (MAF) estimated from the discovery samples.

**Table S4 Proxy SNPs for the GEFOS included in the discovery sample.**

| **Chr** | **GEFOS-SNP** | **A1** | **A2** | **PROXY** | **BP_DIST** | **LD** | ***Gene*** |
| --- | --- | --- | --- | --- | --- | --- | --- |
| 1 | rs12407028 | t | c | rs4233320 | 9265 | 0.94 | *WLS* |
| 1 | rs17482952 | a | g | rs17130550 | 6380 | 0.92 | *WLS* |
| 1 | rs479336 | t | g | rs2586392 | 54620 | 1 | *DNM3* |
| 1 | rs6426749 | c | g | rs7524102 | 13026 | 1 | *ZBTB40* |
| 2 | rs1346004 | a | g | rs3791848 | 12197 | 0.84 | *GALNT3* |
| 2 | rs4233949 | c | g | rs6752877 | 1862 | 0.90 | *SPTBN1* |
| 2 | rs7584262 | t | c | rs11887431 | 16913 | 0.92 | *PKDCC* |
| 3 | rs1026364 | t | g | rs9813630 | 7351 | 0.91 | *KIAA2018* |
| 3 | rs344081 | t | c | rs344081 | 0 | 1 | *LEKR1* |
| 3 | rs430727 | t | c | rs431867 | 8657 | 0.93 | *CTNNB1* |
| 4 | rs3755955 | a | g | rs3755955 | 0 | 1 | *IDUA* |
| 4 | rs6532023 | t | g | rs1471399 | 224 | 1 | *MEPE* |
| 5 | rs1366594 | a | c | rs6885600 | 28332 | 0.81 | *MEF2C* |
| 6 | rs11755164 | t | c | rs6940887 | 2794 | 0.87 | *SUPT3H/RUNX2* |
| 6 | rs4869742 | t | c | rs4870044 | 6339 | 1 | *C6orf97* |
| 6 | rs9466056 | a | g | rs6920208 | 2266 | 1 | *CDKAL1/SOX4* |
| 7 | rs10226308 | a | g | rs17236800 | 5953 | 1 | *RXNDC3* |
| 7 | rs13245690 | a | g | rs13245690 | 0 | 1 | *C7orf58* |
| 7 | rs3801387 | a | g | rs3779381 | 7975 | 0.87 | *WNT16* |
| 7 | rs4727338 | c | g | rs10085588 | 16999 | 1 | *SLC25A13* |
| 7 | rs6959212 | t | c | rs940347 | 17901 | 1 | *STARD3NL* |
| 8 | rs2062377 | a | t | rs4354338 | 8640 | 0.85 | *TNFRSF11B/OPG* |
| 9 | rs7851693 | c | g | rs7466269 | 14743 | 1 | *FUBP3* |
| 10 | rs1373004 | t | g | rs11003047 | 6686 | 0.92 | *MBL2/DKK1* |
| 10 | rs7084921 | t | c | rs11599750 | 8360 | 0.93 | *CPN1* |
| 11 | rs163879 | t | c | rs273558 | 87355 | 0.96 | *DCDC5* |
| 11 | rs3736228 | t | c | rs3736228 | 0 | 1 | *LRP5* |
| 11 | rs7108738 | t | g | rs7128738 | 17856 | 0.95 | *SOX6* |
| 12 | rs2887571 | a | g | rs4283041 | 258 | 1 | *ERC1/WNT5B* |
| 13 | rs9533090 | t | c | rs9594738 | 696 | 0.93 | *TNFSF11/RANKL* |
| 14 | rs11623869 | t | g | rs11623869 | 0 | 1 | *MARK3* |
| 14 | rs1286083 | t | c | rs1286079 | 2383 | 1 | *RPS6KA5* |
| 16 | rs10048146 | a | g | rs10048146 | 0 | 1 | *FOXL1* |
| 16 | rs13336428 | a | g | rs13336428 | 0 | 1 | *C16orf38/CLCN7* |
| 16 | rs1564981 | a | g | rs1564981 | 0 | 1 | *CYLD* |
| 16 | rs4985155 | a | g | rs7200543 | 511 | 0.89 | *NTAN1* |
| 17 | rs227584 | a | c | rs227584 | 0 | 1 | *C17orf53* |
| 17 | rs4790881 | a | c | rs7209460 | 20219 | 1 | *SMG6* |
| 17 | rs4792909 | t | g | rs8068071 | 16045 | 0.90 | *SOST* |
| 17 | rs7217932 | a | g | rs7213040 | 14705 | 0.97 | *SOX9* |
| 18 | rs4796995 | a | g | rs12956554 | 47984 | 0.96 | *FAM210A* |
| 19 | rs10416218 | t | c | rs7259333 | 19999 | 0.90 | *GPATCH1* |
| 20 | rs3790160 | t | c | rs6040061 | 318 | 0.97 | *JAG1* |

**Figures:**

**Figure S1 Principal component analysis calculated for all the Postmenopausal women included in the study.** The colors in the legend denote the ethnic group: EUR, AFR, NAT, MOR and GUAD. Postmenopausal women of Discovery sample (MOR), PC1 and PC2 were calculated using 60, 793 AIMs. Postmenopausal women of replication sample (GUAD), PC1 and PC2 were calculated using 96 AIMs with MAF > 5% and missing call rate < 5%.


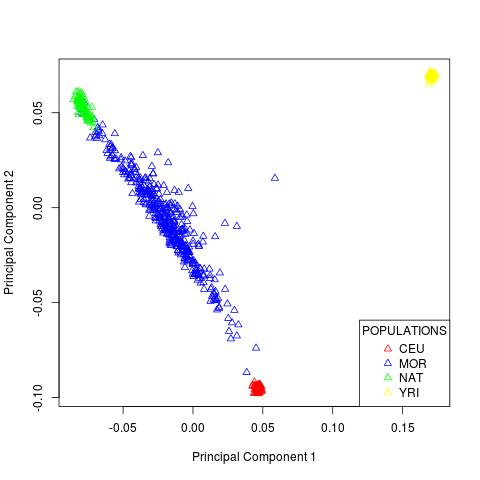

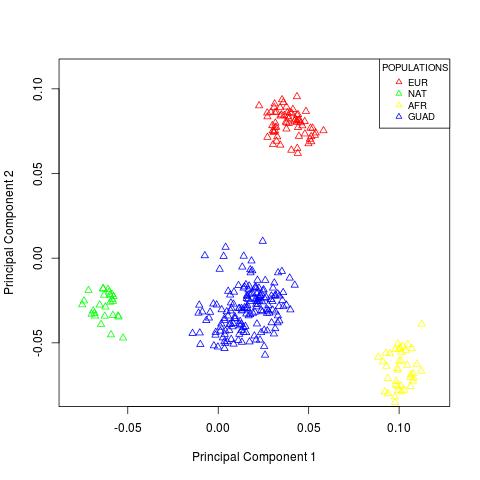


**Figure S2 Manhattan plots of the genome-wide association results of the discovery sample.**


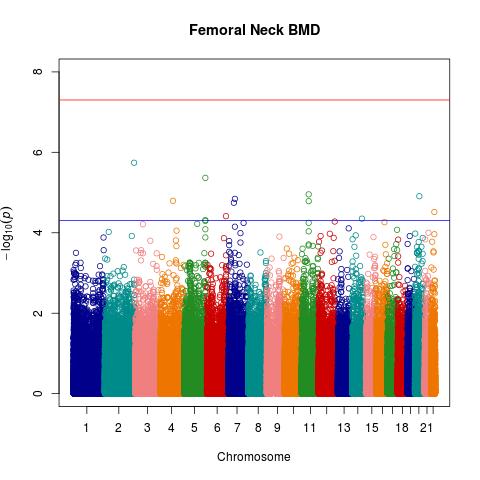


**A**


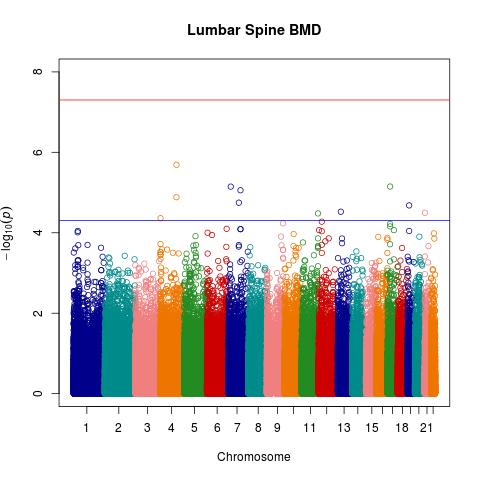


**B**

**Figure S3 Linkage Disequilibrium (LD) plots with the D’ values for the *SLIT3* and *CCDC170* genes.**


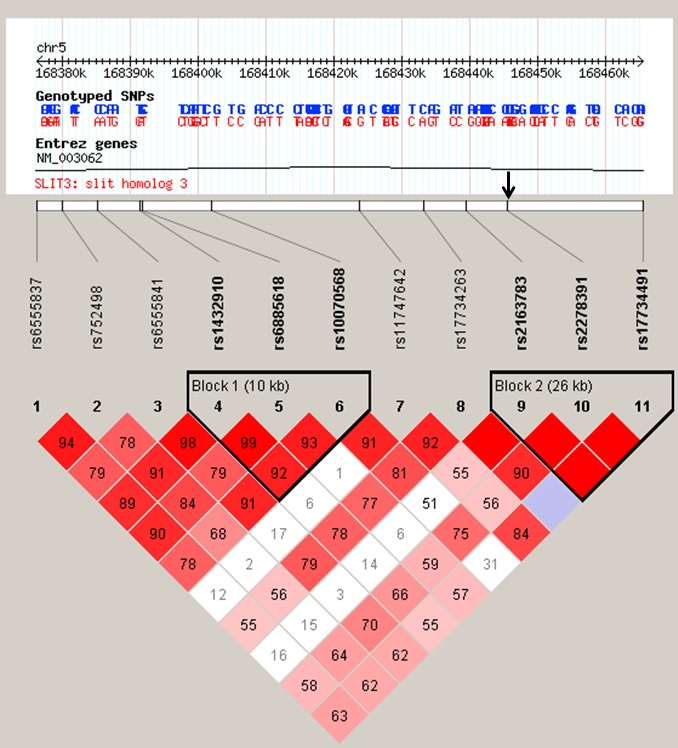


**A**


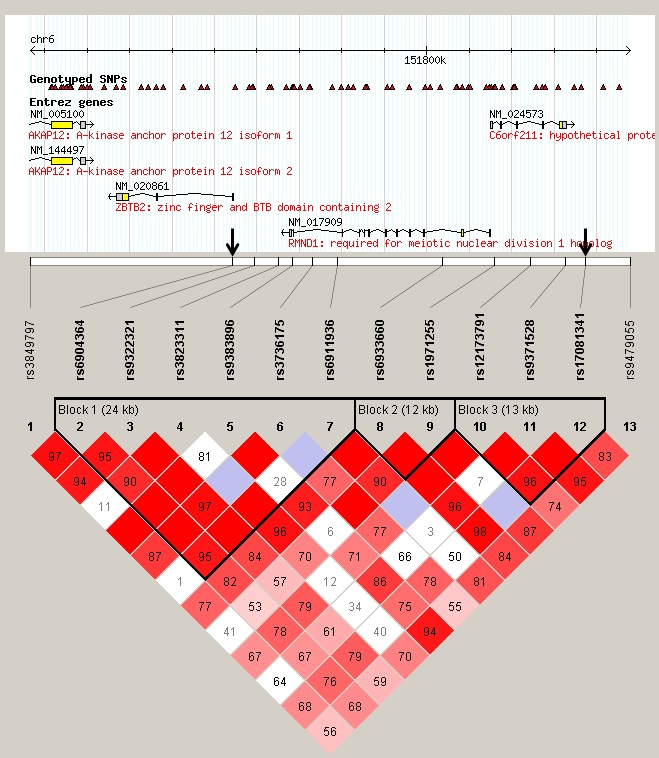


**B**

**Figure S4 Linkage Disequilibrium (LD) plots with the D’ values for the *HDAC9* and *SHFM1* genes.**


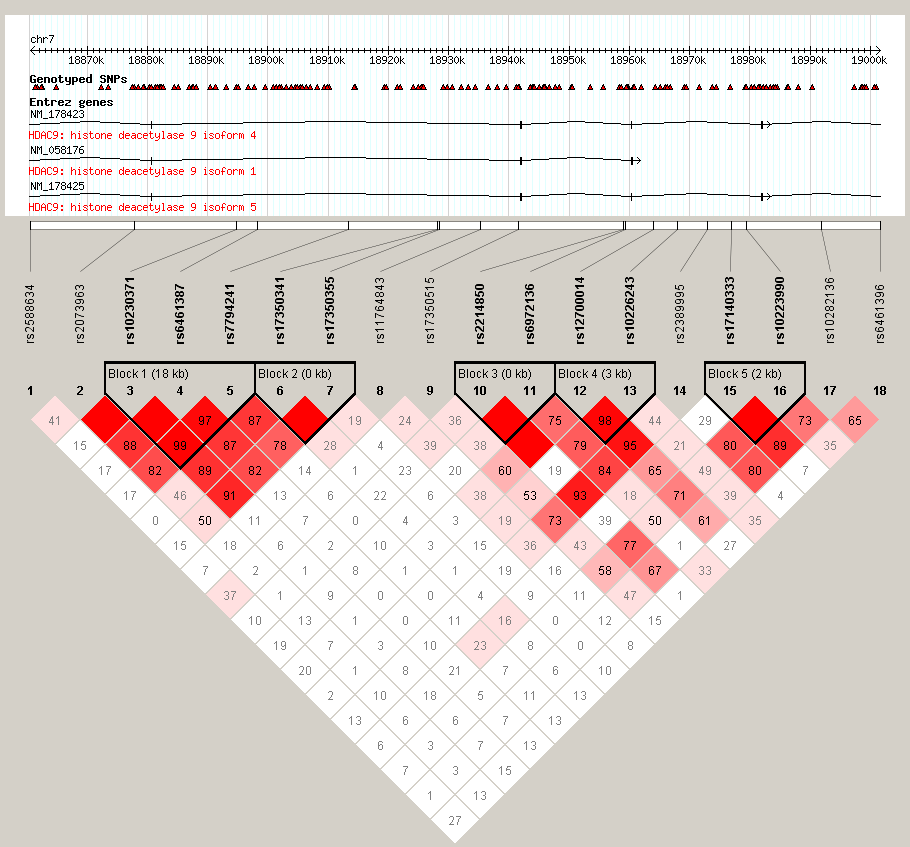


**A**


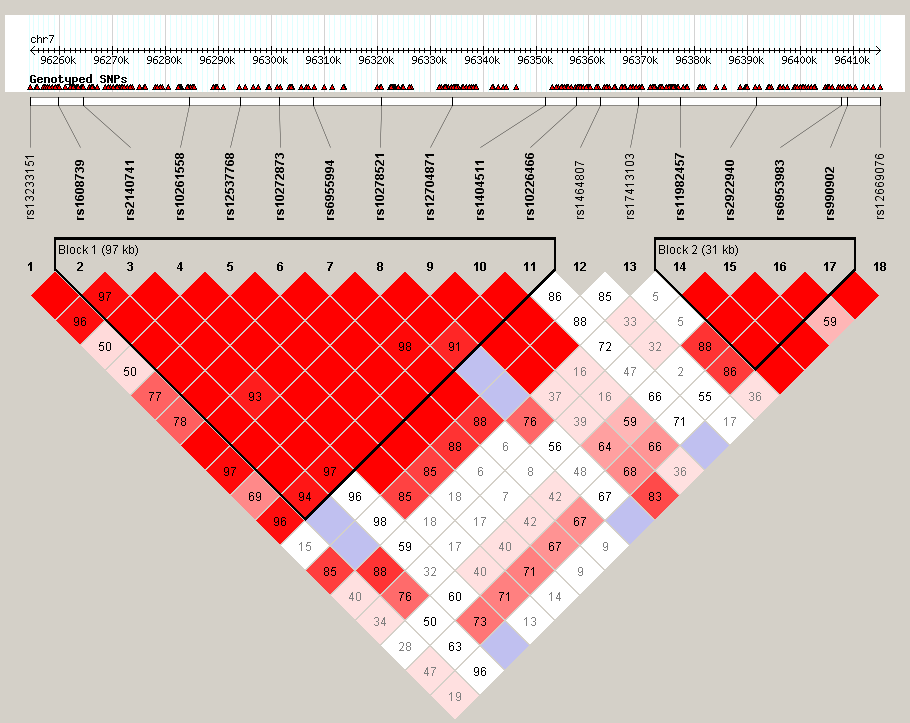


**B**
